# Enhancing astronaut training: effects of transcranial direct current stimulation on manual rendezvous and docking skill acquisition

**DOI:** 10.1186/s12984-025-01737-2

**Published:** 2025-10-27

**Authors:** Jingda Feng, Weifen Huang, Meng Wang, Yusheng Shi, Xuejun Jiao, Yanlei Wang, Yuan Jiang

**Affiliations:** 1 Air Force Medical Center, Air Force Medical University, Beijing, China; 2 Astronaut Center of China, Beijing, China; 3 National Key Laboratory of Human Factors Engineering, Astronaut Center of China, Beijing, China

**Keywords:** Transcranial direct-current stimulation, Manual rendezvous and docking, EEG, Motor learning

## Abstract

**Background:**

Manned spaceflight missions are characterized by significant operational risks, low fault tolerance, and high complexity, necessitating astronauts to achieve exceptional proficiency through training. This study employed a double-blind, randomized, placebo-controlled design to investigate the effects of transcranial direct current stimulation (tDCS) on astronauts’ acquisition of manual rendezvous and docking (RVD) skills. Additionally, electroencephalography (EEG) was utilized to analyze tDCS-induced cortical modulations during manual RVD training.

**Methods:**

A total of 26 participants (tDCS group, *n* = 14; sham group, *n* = 12) completed the experiment. Each participant underwent eight blocks of manual RVD training, with EEG recordings throughout the training. A bilateral M1 montage (anode at C3, cathode at C4) was applied using 1.5 cm ring electrodes, with stimulation lasting 25 min over four training blocks. Learning outcomes for both groups were quantified using composite scores derived from endpoint accuracy parameters and performance metrics related to manual RVD training. Additionally, a skill retention test was administered three weeks after the completion of training.

**Results:**

The tDCS group demonstrated superior performance in skill acquisition compared to the sham group. Significant group × time interactions were observed in both RVD attitude accuracy (*F* = 2.606, *p* = 0.024, Cohen’s d = 0.367) and composite performance scores (*F* = 2.506, *p* = 0.026, Cohen’s d = 0.646), with the tDCS group exhibiting an average performance improvement of 11% over the sham group. Furthermore, retention tests administered three weeks post-training revealed significantly higher scores in the tDCS group (*t* = 2.874, *p* = 0.011, Cohen’s d = 1.189). EEG analysis indicated distinct θ band activity patterns in the right M1 region between the two groups during training [*F*(1,50) = 5.910, *p* = 0.025, *η*² = 0.172], with the tDCS group showing characteristic neural modulation patterns that correlated with enhanced skill learning.

**Conclusion:**

This study demonstrates that bilateral M1 tDCS enhances manual RVD skill acquisition in astronaut training. This technique shows promise for application in astronaut training, potentially improving the efficiency and retention of skill training for space missions.

**Supplementary Information:**

The online version contains supplementary material available at 10.1186/s12984-025-01737-2.

## Introduction

Manned spaceflight tasks are characterized by high risk, low tolerance for errors, complex operations, and stringent precision requirements. Astronauts are required to perform numerous delicate maneuvers during missions, where even minor operational mistakes can lead to significant risks and potentially severe accidents. These challenges necessitate exceptionally high standards for astronaut operational skill training. For instance, the manual rendezvous and docking (RVD) operation skills [1], which every astronaut is required to master, demand precise manual control to achieve the accurate docking of two spacecraft in the complex space environment. This challenging maneuver is often referred to as ‘threading a needle in space’. Additionally, there are numerous other similarly complex operations, such as space robotic arm manipulation and extravehicular activities. However, the extensive curriculum and limited training time present significant challenges for achieving peak operational proficiency. Enhancing the training level of astronaut operational skills within the limited course remains a critical ongoing challenge in the field of astronaut training.

Non-invasive brain stimulation, particularly transcranial direct current stimulation (tDCS), has emerged as a promising tool to accelerate skill acquisition and retention. Research has demonstrated that tDCS can enhance motor skill learning in healthy individuals [2]. The primary focus for modulating motor learning and skill retention has been on unilateral M1 [3–6] (an application of an active electrode over the target area and a return electrode over the contralateral supra orbital area), or bilateral M1 [7–9] (an active electrode over the M1 in one side and a return electrode over the M1 on the other side of the brain). An early study by Nitsche et al. [3] found that applying anodal tDCS to the M1 during training improved online implicit learning of a motor sequence, while stimulation applied to other areas such as the DLPFC, and VLPFC did not enhance learning. A considerable amount of research has also focused on the effects of online tDCS on the acquisition of visuomotor skills for both the upper limbs [10, 11] and lower limbs [12, 13]. For instance, a study by Antal et al. [10] demonstrated that anodal stimulation over the contralateral M1 during the learning process enhanced performance in a visuomotor tracking task. These findings indicated that the secondary network effects of stimulation may be particularly important in dynamic, real-world environments where various cognitive and learning processes constantly interact. Notably, tDCS also enhances complex skills like golf putting [14] and laparoscopic surgery [15], particularly in low-performers [16]. tDCS can also promote the retention of skills learned over multiple days (typically at least three days) of training [17–20]. Reis et al. [21] observed that, compared to a sham group, offline skill gains and retention were significantly enhanced when anodal tDCS was applied to the M1 during five consecutive days of training. This difference between the groups remained when skills were retested three months later, suggesting that these gains had been successfully consolidated and maintained over the long term. Such long-lasting effects may be attributed to the role of transmembrane protein systems like NMDA receptors at the synaptic level [22], mediating processes such as long-term potentiation (LTP) and long-term depression (LTD) [23, 24]. LTP and LTD are critical neurophysiological mechanisms in learning and memory, exerting lasting facilitatory or inhibitory effects on synaptic connections [25].

In contrast to the more widely reported findings, the effective impact of tDCS on the learning of bimanual skills [26] has been very limited. M1 serves as the primary target for tDCS modulation in bimanual skill learning, consistent with the majority of tDCS studies examining unimanual and non-hand motor tasks [25]. Furthermore, the cerebellum [27] and supplementary motor area (SMA) [28] have also been key regions of interest in tDCS research on bimanual learning. Research by Patrick et al. [15] indicated that tDCS to the dominant hemisphere M1 enhanced the learning of unimanual surgical skills but did not significantly promote bimanual skills. Morgan et al. [29] demonstrated that both bilateral M1 tDCS (left anode/right cathode) and SMA tDCS enhanced skill acquisition in Fundamentals of Laparoscopic Surgery (FLS) Peg Transfer task, with bilateral M1 stimulation showing superior efficacy compared to the SMA montage. Pooled evidence from meta-analyses [7, 30] conducted in 2025 and 2020 conclusively demonstrated that bilateral M1 tDCS yields significantly greater improvements in motor learning and performance than unilateral stimulation in healthy individuals, offering critical guidance for subsequent studies.

It is well established that astronautical operations are characterized by exceptional complexity, with most tasks requiring coordinated bimanual control. However, to our knowledge, no studies have systematically examined the effects of tDCS on the acquisition and retention of complex astronautical operational skills. While previous research indicates that tDCS can enhance both the efficiency of skill acquisition and the duration of skill retention, significant variability in its effects has been observed across different studies. Critical issues, including the optimal stimulation sites, current parameters, and modes of stimulation for enhancing astronautical operational skills, remain uncertain and require further in-depth investigation.

This study aimed to investigate the potential of tDCS in enhancing the acquisition of manual RVD skills. Building on existing evidence that tDCS facilitates motor skill learning and performance, we hypothesized that the application of tDCS will improve performance during RVD training. Since the optimal tDCS montage for RVD training has not yet been established, we utilized the bilateral M1 tDCS montage, which has been validated in similar bimanual motor skill learning paradigms [7, 29]. We further believe that this stimulation approach, previously shown to be effective in acquiring surgical skills, will also enhance the efficiency of RVD skill learning.

## Methods

### Participants

A total of 27 healthy male subjects participated in this experimental study. All participants had attained at least a college-level education, were right-handed, free from substance abuse, and had no history of psychiatric illness. Prior to the experiment, none of the participants had previous experience with manual RVD or any form of spacecraft flight operation. Due to the inclusion of tDCS in the experiment, a rigorous screening of each subject’s medical history was conducted, which assessed conditions such as brain tumors, a family history of epilepsy, previous head surgery, and the presence of metallic implants. Participants who met the inclusion criteria provided written informed consent. Before the study commenced, participants were randomly assigned to either the tDCS group or the sham group using computer-generated randomization. They were instructed to ensure they received at least 8 h of sleep the night before the experiment and to abstain from alcohol the day before, as well as from caffeine and nicotine on the day of the experiment. This double-blind, randomized controlled trial received ethical approval from the Ethics Committee of the China Astronaut Research and Training Center.

### Experimental design and procedure

The experiment was divided into three phases: the preparation phase, the implementation phase, and the post-experimental interview.

The primary focus of the preparation phase was to instruct participants on the task and to fit them with the experimental equipment. This instruction took approximately 20 min. Participants began by independently reading the operation manual. Following this, a primary investigator provided a standardized explanation of the principles of RVD and demonstrated the operational methods. Following the demonstration, participants engaged in simple operational exercises to familiarize themselves with the handle polarity, but they did not practice actual RVD tasks. Once the instruction was complete, participants were fitted with the tDCS device and EEG recording equipment. Conductive paste was applied to minimize contact impedance between the equipment and the scalp, thus completing all pre-experimental preparations.

**Fig. 1:**
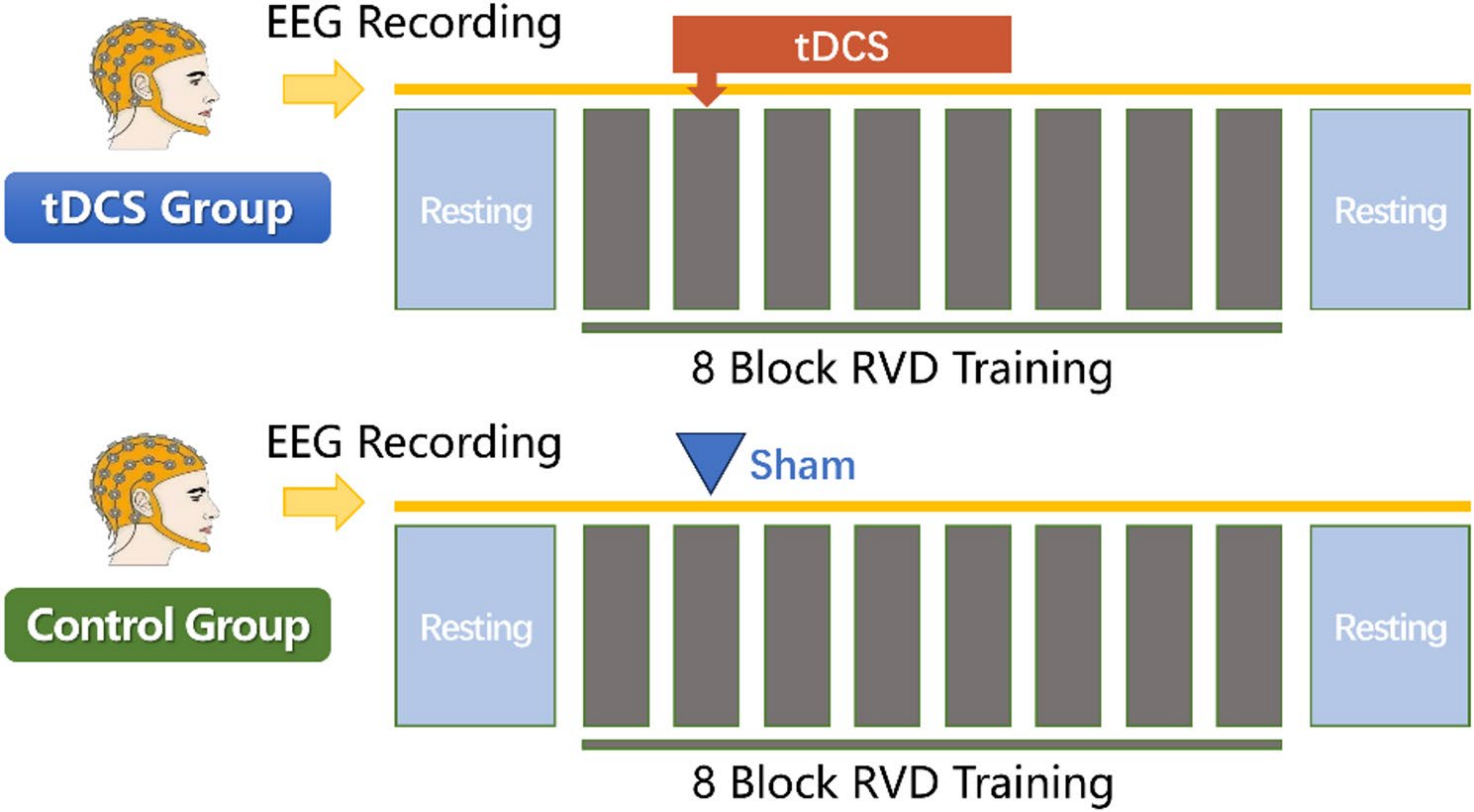
Flowchart of the experimental design

During the implementation phase, participants were required to perform RVD training across eight training blocks. Each block had different initial settings but maintained the same level of difficulty, with each block lasting 6 to 8 min. After each block, participants completed the NASA-TLX mental workload scale to assess the cognitive load experienced during the previous RVD training block. The first block served as a baseline skill assessment, with tDCS being initiated at the beginning of the second training block and continuing for 25 min, concluding after the fifth training block. Behavioral performance data and EEG data were recorded throughout the training. Due to significant interference from tDCS on EEG collection, only the EEG data from the first and eighth training blocks were included in the offline analysis. The experimental procedure is illustrated in Fig. 1.

In the post-experimental interview phase, participants took part in a brief interview designed to gather their subjective experiences related to tDCS and any additional feedback.

### Experimental task

**Fig. 2:**
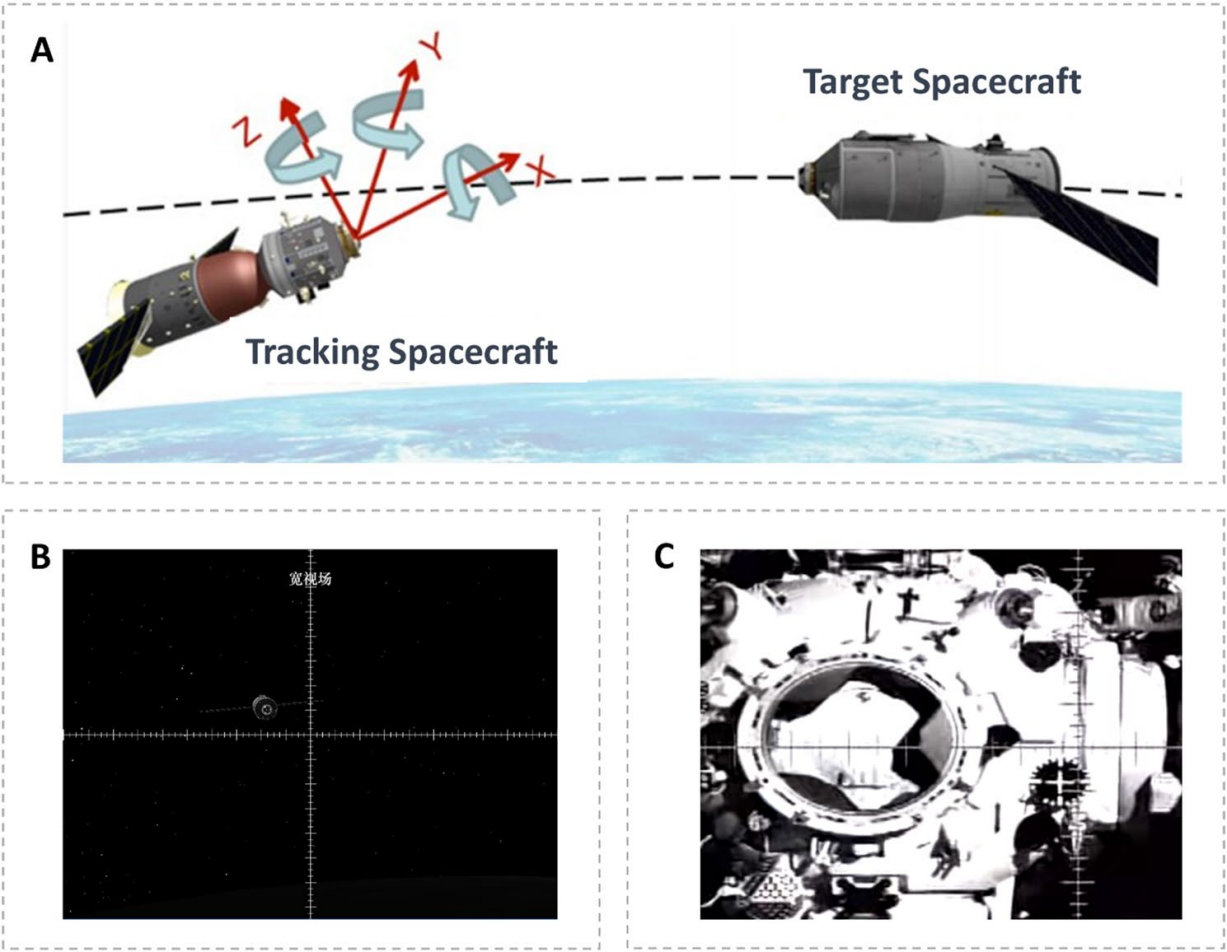
Schematic diagram of manual control rendezvous and docking mission

The experimental task selected for this study was a simulation of spacecraft manual rendezvous and docking. It utilized a desktop manual RVD training platform developed by the China Astronaut Research and Training Center. Manual RVD involves utilizing onboard equipment, such as television cameras, to monitor the relative positions and orientations of the two spacecraft while manually operating the controls to successfully complete the docking process. Figure 2 illustrates the RVD task. In A, the spatial relationship between the chasing spacecraft and the target spacecraft throughout the RVD process is depicted. B and C show profiles of the target spacecraft from the perspective of the chasing spacecraft at both long and short distances, respectively, along with the docking targets. During the manual RVD process, astronauts must make real-time judgments about the position and orientation of the chasing spacecraft based on the relative positions displayed on the screen. By manipulating the translation and attitude handles, the astronaut adjusts the six degrees of freedom of the chasing spacecraft’s motion to meet the stringent docking requirements for position, velocity, attitude, and angular velocity. The left handle controls translation, allowing adjustments to the relative displacement along the three spatial axes (x, y, and z), while the right handle manages attitude, enabling adjustments to the relative roll, pitch, and yaw angle deviations.

The experimental task involved a manual RVD operation with fixed difficulty. The initial relative velocity of the spacecraft along the x-axis was set to 2 m/s, while the initial relative velocities along the y-axis and z-axis were set at 0.2 m/s. During the training process, participants were instructed not to adjust the spacecraft’s x-axis velocity, and the docking completion time was not evaluated for performance. The training task included initial position and attitude deviations. Specifically, the initial distance between the target spacecraft and the chasing spacecraft along the x-axis was set to 70 m, with horizontal (y-axis) and vertical (z-axis) position deviations of 5 m each. Furthermore, the target and chasing spacecraft had relative pitch, yaw, and roll angles of 5° each. Participants were required to use the translation control handle (left handle) to adjust the position deviations and the attitude control handle (right handle) to correct the attitude deviations.

### tDCS specifications

The tDCS device used was DC-Stimulator (NeuroConn, Germany). The device utilized ring-shaped silver chloride electrodes, each with a diameter of 1.5 cm, affixed to a standard EEG cap arranged according to the 10/10 system. Conductive paste was applied to ensure a reliable connection between the stimulator electrodes and the scalp, with electrode impedance maintained below 5 kΩ. A ramp-up period of 30 s was implemented to gradually increase the current from 0 mA to 2 mA. After this initial ramp-up, the current for the tDCS group was sustained at 2 mA for 25 min, followed by a gradual ramp-down to 0 mA over another 30 s. In contrast, the sham group experienced a similar ramp-up over 30 s, but the current was immediately decreased back to 0 mA over an additional 30 s and maintained at that level until the end of the stimulation. Throughout the entire stimulation process, continuous monitoring was conducted to detect any increases in contact impedance between the electrodes and the scalp. No participants withdrew from the experiment due to intolerance to the tDCS intensity.

**Fig. 3:**
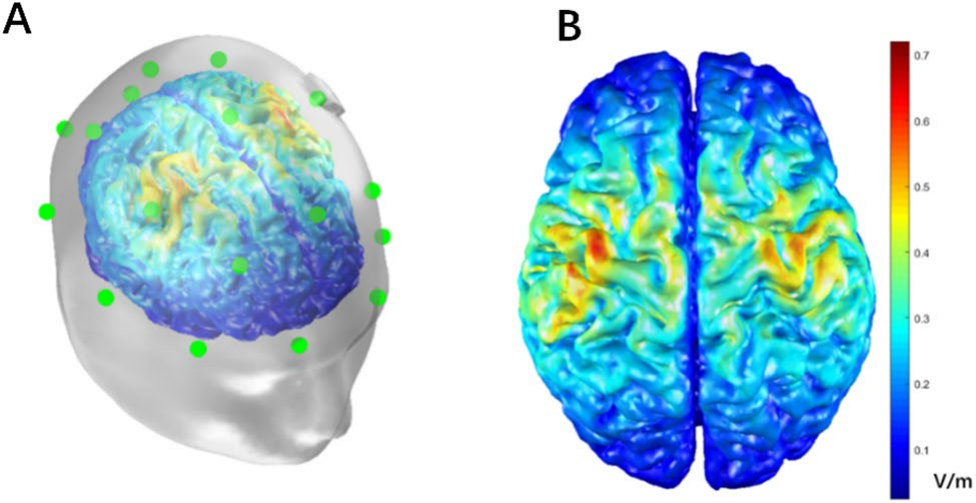
Simulation of cortical electric field strength.** A** The transparent tissue segmentation of the scalp, skull, and cortex.** B** The underlying electric field distribution on a generic T1-weighted MRI, with the color scale bar representing the electric field strength

Based on the experimental hypotheses and theoretical foundations presented earlier, we employed a bilateral primary motor cortex montage. The anode was positioned over the left M1 at the C3 electrode site, while the cathode was placed over the right M1 at the C4 electrode site, following the 10/10 EEG system. To simulate the electric field intensity of tDCS, we utilized the MATLAB-based COMETS v2.0 open-source toolkit [31]. The simulation results indicated that the electric field effectively encompassed the hand motor area of the primary motor cortex. Figure 3A displays the transparent tissue segmentation of the scalp, skull, and cortex, along with the corresponding electric field. Figure 3B shows the electric field distribution in the cortex, with the color scale bar representing the electric field magnitude, in units of volts per meter (V/m).

### Behavioral data processing

The task performance data for the manual RVD training were automatically recorded and stored by a desktop simulation RVD platform. The performance data primarily included the time taken to complete RVD (spacecraft time), propellant consumption, relative position at the time of docking (x, y, and z axes), relative velocity at docking, relative roll angle at docking, relative pitch angle at docking, and relative yaw angle at docking, among others.

Propellant consumption, accuracy at the time of docking completion, and the time required to complete docking are key indicators for assessing the performance of manual RVD training. In this study, to better control the research variables, a uniform docking speed was maintained throughout all training sessions. Consequently, the time taken to complete docking and the relative velocity of docking were not used as assessment indicators. Based on the regression model established by Wang Meng et al. [32], it was found that each indicator contributes approximately equally to overall performance, with propellant consumption, docking attitude, and position accuracy all exhibiting a negative correlation with the final score. In this paper, the position accuracy indicator is defined as the sum of the absolute values of the docking positions across the three axes, while the attitude accuracy indicator is defined as the sum of the absolute values of the roll, pitch, and yaw angles of the RVD. To facilitate comparison, the indicators for propellant consumption, position accuracy, and attitude accuracy are normalized and scaled to a common range of 0 to 100 points using the max-min standardization method, enabling the calculation of a composite operation score. Since all three indicators have a negative correlation with training proficiency, the following formula is employed, wherein the score for each indicator is subtracted from 100:

1 $$Score=\frac{1}{3}\left[ {\left( {100 - fu} \right)+\left( {100 - dist} \right)+\left( {100 - dire} \right)} \right]$$

In the formula, (*fu*) represents the normalized propellant consumption, (*dist*) represents the normalized position accuracy, and (*dire*) represents the normalized attitude accuracy. The final composite score ranges from 0 to 100 points, with higher scores indicating better task performance.

Given the high complexity and operational difficulty of the manual RVD task, participants with no prior experience may encounter individual trials during training where operational and judgment errors lead to significant deviations from the target spacecraft. To mitigate the impact of extreme outlier data on the results, weconducted outlier detection on all behavioral outcomes using the Generalized Extreme Studentized Deviate (GESD) algorithm prior to statistical analysis. This method relies on hypothesis testing and iteratively removes outliers, proving effective in scenarios with overlapping multiple outliers [33]. Among the 1,638 performance data points, 31 outliers were identified. Median interpolation from the data of the same training group was employed to replace these outliers, which accounted for 1.89% of the total dataset.

### EEG signal acquisition and analysis

EEG activity during the task was recorded using the eego™ mylab recording system (ANT neuro, Netherlands), 64 electrodes were fixed on the elastic cap with electrode positions configured according to the international 10/10 system. The channel impedance was less than 10 kΩ and the EEG signal sampling rate was 1000 Hz. EEG data were preprocessed offline using EEGLAB (2021.0) [34], running in the Matlab environment. The signals were first down-sampled to 500 Hz and digitally filtered using a 0.1–30 Hz Butterworth bandpass filter, and then the signals were re-referenced to the bilateral mastoid electrodes (M1 and M2 leads). Artifacts were eliminated using infomax independent component analysis (ICA) methods [35] of EEGLAB. Components containing blinks, ocular drift, muscle artifacts, electro cardiographic artifacts, or head movement were identified by topography and subtracted from the data. The preprocessed EEG data were segmented into 2-second data segments with no overlap between adjacent segments. Finally, a data quality check was conducted. Epochs were rejected on the basis of amplitude extreme values exceeding ± 80 µV.

**Fig. 4:**
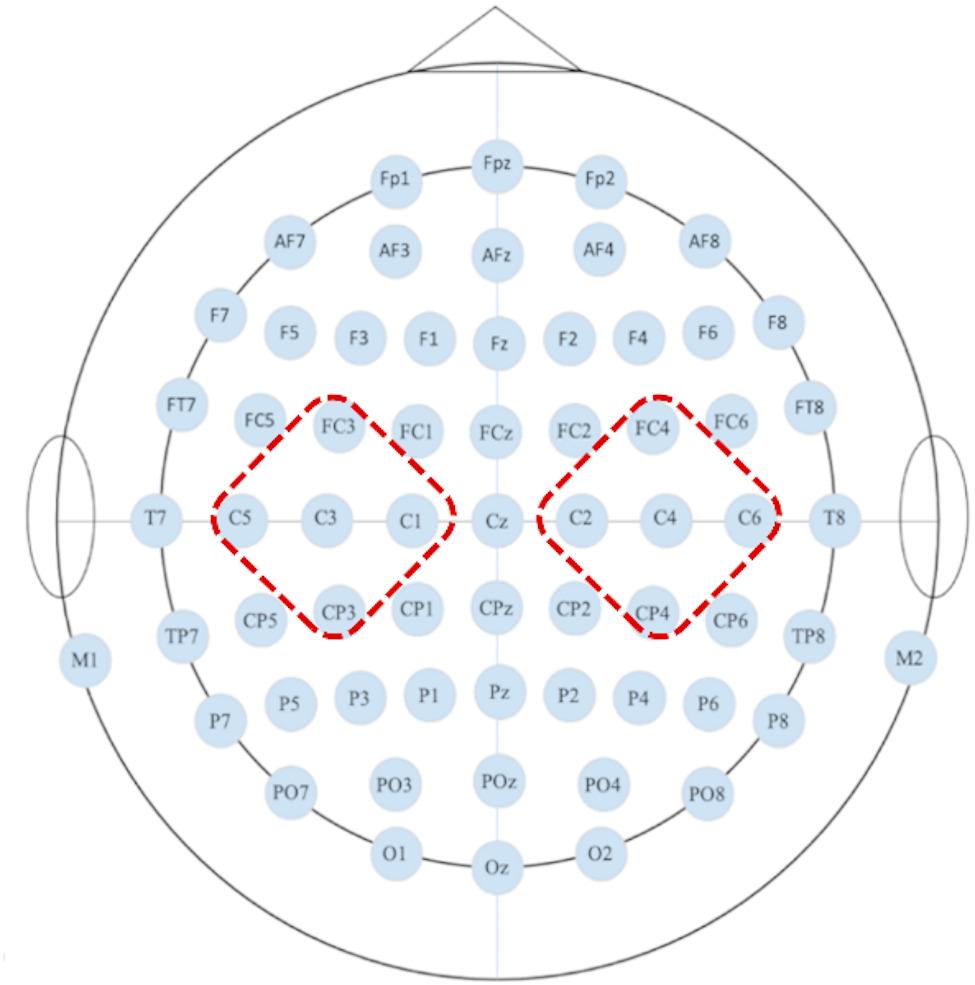
The distribution of EEG electrodes and tDCS target areas

The Welch method was employed to estimate the power spectrum of EEG data across three frequency bands: theta band (θ,4–8 Hz), alpha band (α,8–13 Hz), and beta band (β,13–30 Hz). This analysis specifically examined changes in the power spectra of θ, α, and β waves within the target regions of tDCS (bilateral M1). The left M1 positions analyzed were C1, C3, C5, FC3, and CP3, while the right M1 positions included C2, C4, C6, FC4, and CP4. The distribution of EEG electrodes and the electrode positions analyzed in the study are shown in Fig. 4.

### Statistical analysis

The baseline characteristics of the subjects were analyzed using an independent samples t-test. Learning curves were calculated to evaluate changes in overall performance throughout the training process.

The Shapiro-Wilk test was utilized to assess the distribution of the learning curve data. A linear mixed effects model (LME) was employed to analyze the effects of tDCS on the learning curves, with individual subjects treated as a random factor and training blocks and subject groupings treated as fixed effects. The reported main and interaction effects indicate the statistical outcomes of comparisons between training groups over the entire training period. The residual covariance with a compound symmetry structure was modeled using repeated effects from training group blocks. In addition to analyzing the main effects of training blocks and groups, the interaction effect between training blocks and groups was also included (with a significance level of *p* < 0.05 considered statistically significant). Cohen’s d was computed to quantify the effect size, providing a standardized measure of the magnitude of the difference between groups.

To evaluate retention test scores after three weeks, an independent samples t-test was conducted, complemented by Levene’s test to assess the homogeneity of variances in task performance between the two groups. In instances where the assumption of equal variances was violated, the Satterthwaite approximation t-test was used to adjust the degrees of freedom.

A two-way repeated-measures analysis of variance (ANOVA) with within-subjects factor Test (pre-training, post-training) and between-subjects factor Group (tDCS group, sham group) was performed to examine the power spectrum characteristics of EEG signals in tDCS target regions. Bonferroni-corrected post hoc tests were employed for pairwise group comparisons. The statistical effect sizes were reported through η². The analysis focused on time × group interaction effects and between-group differences in spontaneous EEG power spectra. All statistical analyses were conducted using SPSS (Version 25; IBM Corp., Armonk, NY, USA) and MATLAB (Version 2018a; MathWorks, Natick, MA, USA), with linear mixed-effects models fitted using the fitlme function in MATLAB.

## Results

### Demographics

One participant from the sham group withdrew during the experiment, resulting in a final effective sample size of 26 participants (14 in the tDCS group and 12 in the sham group). All participants were recruited from staff members at the China Astronaut Research and Training Center and students from China Agricultural University. The sample consisted exclusively of right-handed male participants. No significant between-group differences were observed in terms of age or years of education (*p* > 0.05). Moreover, there was no significant difference in the baseline skill test scores (training block 1) between the two groups of participants (*t* = 0.329, *p* = 0.745) (Table 1).

**Table 1:** Baseline characteristics

	tDCS group (*n* = 14)	Sham group (*n* = 12)	*p*-value
Age in years (SD)	29.36 (4.60)	28.50 (6.58)	0.70
Year of education (SD)	14.07 (2.06)	14.50 (1.45)	0.55
Male (%)	14 (100%)	12 (100%)	
The Han ethnic (%)	13 (92.9%)	11 (91.7%)	
Right-handed(%)	1 4(100%)	12 (100%)	
Baseline test score (training block 1)	47.6 7(11.07)	49.69 (19.73)	0.745

### Position and attitude accuracy of RVD

As training progressed, both groups exhibited significant improvements in position accuracy during docking. The number of training blocks had a considerable impact on docking position accuracy (*F* = 11.833, *p* < 0.001, Cohen’s d = 1.513). Specifically, the average deviation in docking position decreased from 0.79 m in the 1st training block to 0.34 m by the 8th block. Moreover, the effectiveness of the training was indicated by a gradual reduction in the standard deviation of position accuracy, which decreased from 0.31 m in the 1st block to 0.18 m in the 8th block. A calculated percentage improvement in training scores revealed that the tDCS group experienced greater gains, with an average position accuracy improvement of 65%, compared to 48% for the sham group.However, the interaction between group and training blocks did not reach statistical significance (*F* = 1.542, *p* = 0.178, Cohen’s d = 0.559). Similarly, the between-group main effect failed to reach statistical significance (*F* = 3.018, *p* = 0.095, Cohen’s d = 0.440).

Attitude control accuracy exhibited comparable learning patterns throughout the training progression. Both groups achieved significant improvements in attitude accuracy, with a notable main effect of training blocks on this metric (*F* = 10.069, *p* < 0.001, Cohen’s d = 1.748). The average deviation in attitude accuracy decreased from 6.66° in the 1st training block to 2.12° by the 8th block, and the standard deviation of attitude accuracy also significantly reduced from 4.11° in the 1st block to 1.16° in the 8th block. No significant between-group main effect was observed (*F* = 0.440, *p* = 0.510, Cohen’s d = 0.086). However, we identified a significant group × training block interaction (*F* = 2.606, *p* = 0.024, Cohen’s d = 0.367), suggesting that tDCS accelerated the acquisition of spacecraft attitude control skills. The tDCS group demonstrated superior learning gains, showing a 75% improvement in attitude accuracy versus 59% for sham controls.

**Fig. 5:**
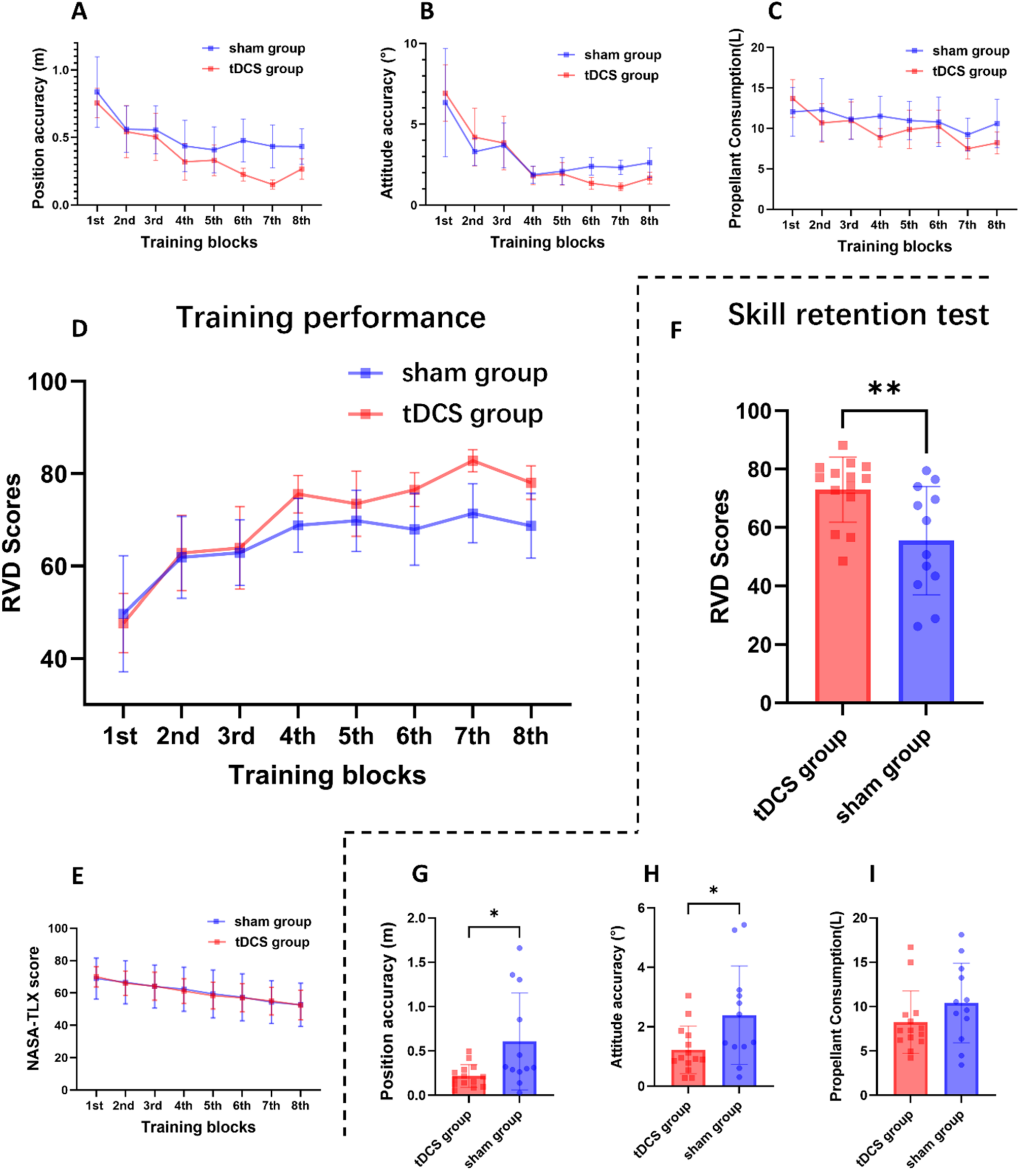
Training performance and skill retention.** A** Learning curve of position accuracy across training blocks;** B** Learning curve of attitude accuracy across training blocks;** C** Learning curve of propellant consumption across training blocks;** D** Learning curve of RVD score across training blocks;** E** Learning curve of NASA-TLX score across training blocks;** F** RVD score of skill retention test;** G** Propellant consumption of skill retention test;** H** Attitude accuracy of skill retention test;** I** Position accuracy of skill retention test

Figure 5 illustrates the training performance of the participants, with** A** illustrating the learning curve of position accuracy across training blocks and** B** illustrating the learning curve of attitude accuracy across training blocks. The detailed data of all training blocks are provided in the supplementary materials (Table S1)

### Propellant consumption

As training progressed, both groups of subjects exhibited a significant reduction in propellant consumption (*F* = 6.339, *p* < 0.001, Cohen’s d = 0.889). The main effect of group (*F* = 1.050, *p* = 0.315, Cohen’s d = 0.263) and the interaction effect between group and training blocks (*F* = 1.437, *p* = 0.216, Cohen’s d = 0.589) were not significant. Figure 5C illustrates the learning curves of propellant consumption for both groups.

### RVD composite scores

The RVD composite scores provided a comprehensive assessment of participants’ overall performance. Both groups exhibited significant performance enhancement throughout the training, as reflected in their composite scores (*F* = 18.800, *p* < 0.001, Cohen’s d = 1.739). Mean scores improved substantially from 48.6 ± 15.4 points from initial training to 73.7 ± 9.8 points by the final session. This improvement was accompanied by a marked reduction in score variability (SD decreasing from 15.4 to 9.8 points), suggesting more consistent operational proficiency across participants. A significant interaction effect between groups and training blocks was identified throughout the training period (*F* = 2.506, *p* = 0.026, Cohen’s d = 0.646). The tDCS group achieved an average score improvement of 30%, compared to only 19% for the sham group. No significant between-group differences were observed in the main analysis (*F* = 2.674, *p* = 0.114, Cohen’s d = 0.344). Figure 5D shows the learning curve of RVD score.

### Mental workload

The mental workload of subjects in both groups gradually decreased throughout the training (*F* = 35.169, *p* < 0.001, Cohen’s d = 1.418). There were no significant differences in cognitive load changes between the two groups (*F* = 0.001, *p* = 0.972, Cohen’s d = 0.012), and the interaction effect between subject groups and training blocks was not statistically significant (*F* = 0.184, *p* = 0.672, Cohen’s d = 0.008). The changes in mental workload among participants are illustrated in Fig. 5E.

### Skill retention testing

Only the propellant consumption data satisfied the assumption of homogeneity of variance during the retention test. There were no significant differences in propellant consumption between the tDCS group and the sham group during the retention test (*t* = – 1.351, *p* = 0.191, Cohen’s d = – 0.542). However, the results of the Satterthwaite-corrected t-tests revealed that the tDCS group exhibited significantly higher position accuracy (*t* = – 2.420, *p* = 0.032, Cohen’s d = -1.023) and attitude accuracy (*t* = – 2.347, *p* = 0.015, Cohen’s d = – 0.939) compared to the sham group. Moreover, the composite score for the tDCS group was significantly higher than the sham group’s (*t* = 2.874, *p* = 0.011, Cohen’s d = 1.189), as illustrated in Fig. 5F–I. The detailed data of all retention tests are provided in the supplementary materials (Table S2).

### EEG power

**Fig. 6:**
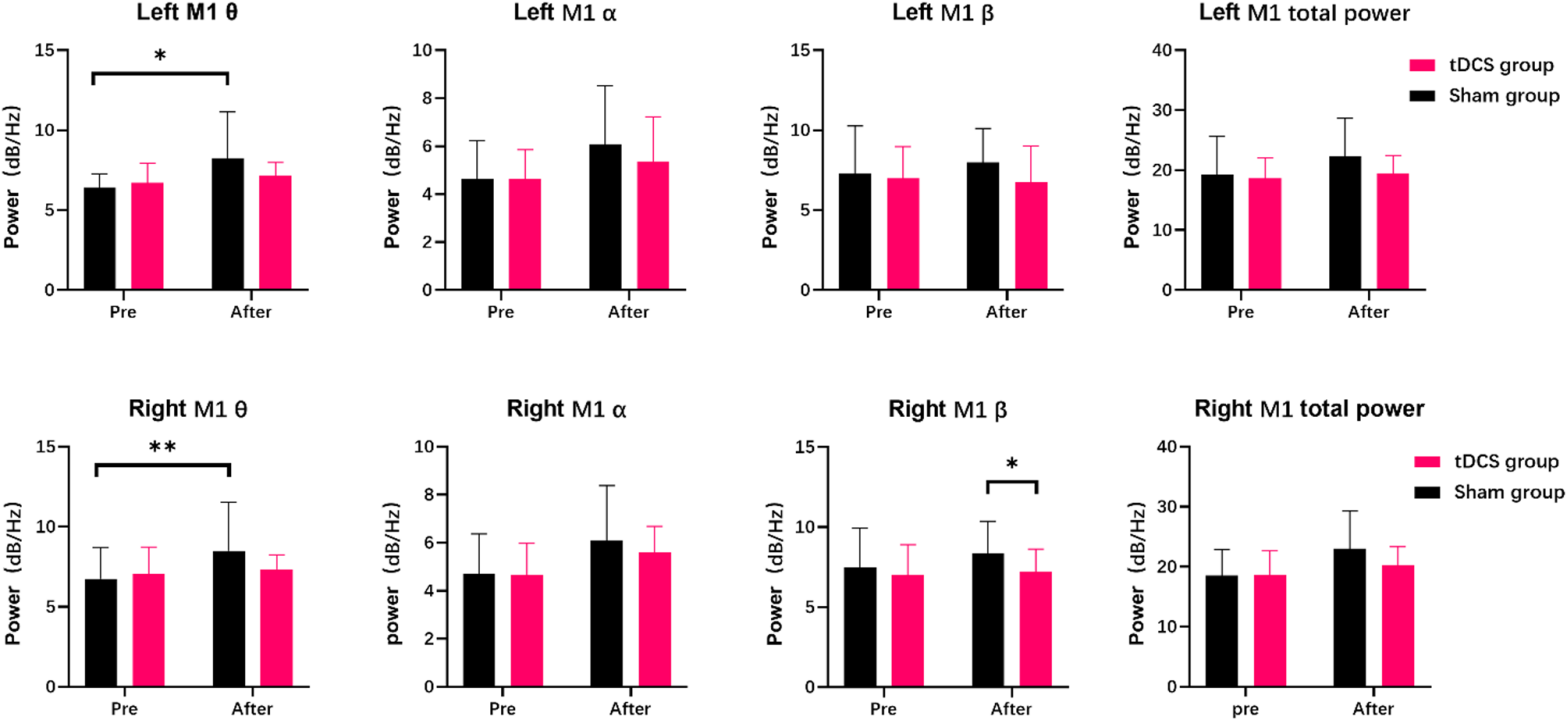
EEG power bar chart before and after RVD skill learning

The EEG power of the bilateral target areas (left and right M1) was analyzed during the experiment, as illustrated in Fig. 6. Among all statistical results, only the theta power exhibited a significant change. Training led to a notable increase in theta power for the left M1 [*F*(1,50) = 5.910, *p* = 0.025, *η*² = 0.172] and the right M1 [*F*(1,50) = 8.576, *p* = 0.008, *η*² = 0.300]. More importantly, the theta power of the right M1 showed a significant interaction effect between training stages and participant groups [*F*(1,48) = 5.410, *p* = 0.031, *η*² = 0.213]. The left M1 theta power also approached significance [*F*(1,48) = 4.151, *p* = 0.055, *η*² = 0.172]. No significant differences were observed in other frequency bands or total power between training stages and participant groups, with detailed results presented in the supplementary materials (Table S3).

Post-hoc pairwise comparisons with Bonferroni correction revealed that when comparing EEG power between the early and later stages of training, the sham group demonstrated a significant increase in theta power at both the left M1 (*t* = 3.146, *p* = 0.012) and the right M1 (*t* = 3.503, *p* = 0.007). In contrast, the tDCS group did not show significant changes in theta power at the left M1 (*t* = 0.283, *p* = 0.782) or the right M1 (*t* = 0.453, *p* = 0.660).

### tDCS tolerability

No serious adverse events were reported during the experiment. Overall, participants’ self-reported sensations did not differ significantly between groups, with most describing the experience as mild tingling or itching. In the tDCS group, 6 participants reported tingling, 3 reported itching, and 5 reported no noticeable sensations, while in the sham group, these numbers were 3, 4, and 5, respectively. The comparable proportions of participants reporting either tingling or itching sensations (64.3% in the tDCS group vs. 58.3% in the sham group) suggest that subjective experiences were similar between the two conditions.

## Discussion

This study aimed to investigate the effects of tDCS on the performance of a manual RVD task requiring coordinated bimanual control. While prior research has demonstrated that tDCS enhances motor skill learning, these studies have predominantly examined unilateral stimulation paradigms in simpler unimanual tasks. Our findings reveal that tDCS combined with RVD training produced significantly greater performance improvements during acquisition compared to sham stimulation, with these benefits persisting at the 3-week retention test. These results provide empirical evidence that tDCS effectively facilitates the learning of complex bimanual RVD skills.

The behavioral results demonstrate that bilateral tDCS significantly improved training performance in the RVD task. Specifically, the active stimulation group showed greater enhancements in both positional and attitudinal docking accuracy compared to the sham control group. However, we found no significant differences in propellant consumption between the groups, a finding that may initially seem counterintuitive, as fuel efficiency is generally regarded as a more sensitive indicator of operator expertise among skilled performers. This result likely reflects the participants’ current skill level, as they had not yet achieved the proficiency threshold at which propellant usage becomes a discriminating measure of performance. The tDCS group demonstrated significantly faster improvements in RVD composite scores compared to the sham group, supporting our hypothesis that tDCS enhances RVD skill acquisition. While NASA-TLX measurements indicated a significant reduction in mental workload during training for both groups, no significant differences in subjective mental workload between the groups were observed. The three-week retention test revealed persistent performance differences between groups, particularly in position and attitude accuracy during docking maneuvers.

Baseline deviations in both position and attitude accuracy were unavoidable, and subjects exhibited variability in their scores during baseline testing. However, the baseline differences between the two groups were noteworthy: the tDCS group had lower position accuracy, while the sham group showed lower attitude accuracy. Overall scores were comparable between the two groups. Given that we achieved an average sample size of 13 subjects per group, these baseline variations fall within an acceptable range. A review by Buch et al. [25] on tDCS and motor skill learning reported that the average sample size in 39 related studies was 13.4 subjects per group, suggesting that our study aligns well with typical standards in this field.

Current evidence suggests that bilateral tDCS serves as an effective montage for facilitating motor skill acquisition [30]. Traditional models suggest that anodal tDCS increases cortical excitability, while cathodal stimulation decreases it. However, research has shown that cathodal stimulation at intensities of 2 mA or higher can induce nonlinear neurophysiological effects [36]. Importantly, current intensity is a crucial factor in determining excitability patterns. Specifically, 2 mA stimulation has been found to produce a net increase in excitability beneath both anodal and cathodal electrodes [36, 37]. These findings challenge the simplistic “cathodal inhibition/anodal excitation” dichotomy, revealing instead that tDCS effects depend on multiple parameters including current intensity, stimulation duration, and region-specific cortical properties [7]. At higher current levels, cathodal stimulation may activate inhibitory interneurons, thereby enhancing overall cortical output through disinhibition mechanisms. Moreover, bilateral 2 mA tDCS applied over M1 has been demonstrated to enhance functional connectivity within motor networks [38]. The inclusion of contralateral, opposite-polarity stimulation further diminishes interhemispheric inhibition (IHI) and improves bimanual coordination [39, 40], providing strong empirical support for the selection of this montage in the present study.

The learning of bilateral and unilateral skills involves distinctly different neural mechanisms. Electric field simulations revealed that the bilateral tDCS montage produced a more focused electric field across the entire motor area compared to unilateral stimulation, which resulted in a broader distribution of the electric field across the right primary motor cortex and frontal regions. This tDCS montage aimed to modulate and enhance the effects of IHI within the primary motor cortex. The generation and control of complex bimanual movements engage various cortical and subcortical regions [41, 42]. Research has shown that the coordinated movements of both hands rely on unique interactions between the cortices of both hemispheres, as well as subcortical regions, all regulated through the corpus callosum. Adults with acquired damage to the corpus callosum often face significant challenges when performing bimanual tasks [42]. Thus, IHI between the primary motor cortices, mediated by the corpus callosum, is recognized as a crucial mechanism for executing bimanual movements. In the manual RVD task, the left hand controls the translation of the spacecraft, while the right hand manages its attitude. Moreover, maintaining the target spacecraft in the center of the field of view often requires simultaneous adjustments in both translation and attitude. Consequently, manual RVD serves as a prime example of differential actions performed concurrently by both hands. In a recent study, researchers examined the impact of applying tDCS to the dominant hemisphere during laparoscopic surgical pattern cutting and peg transfer training tasks. They found that subjects who received tDCS demonstrated greater skill improvement in the pattern cutting task compared to those who received sham stimulation. However, tDCS did not show a significant intervention effect on the peg transfer training task [15]. It is important to note that while both tasks involve the use of both hands, they differ significantly in their demands. The pattern cutting task is primarily unilateral, as the non-dominant hand mainly serves as a stabilizer with minimal movement involved. In contrast, the peg transfer task is a purely bilateral task that requires equal engagement of both hands.

θ waves are closely associated with cognitive functions such as learning, memory, attention, and navigation [43, 44]. During the early stages of skill learning, the cortex needs to process and integrate increased amounts of sensory and motor information [45], which is reflected in elevated θ wave activity. Typically, θ power displays a distinct pattern throughout the skill acquisition process: it initially increases and then decreases [46]. At the onset of skill learning, heightened cognitive load leads to elevated θ activity. As individuals progress and gain proficiency, the execution of the skill transitions from a controlled, effortful process to an automated one, resulting in a gradual decline in θ power. This decline signifies a reduced requirement for cognitive resources during task performance [47]. Therefore, increased θ power serves as a reliable biomarker for identifying the early stages of skill learning [46, 47]. In our study, we observed a significant group-by-time interaction effect in θ power. After eight training blocks, the tDCS group exhibited lower θ activity compared to the sham group. This finding suggests that the tDCS group progressed through the early stages of skill acquisition more quickly and transitioned into the automation phase sooner. The shift in θ power serves as a key indicator of accelerated skill learning in the tDCS group.

It is generally believed that anodal tDCS enhances the excitability of neurons in the targeted brain region, which is associated with an increase in EEG power [48–50]. This effect may be related to improvements in cognitive functions such as learning andmemory [49]. However, with bilateral tDCS, both anodal and cathodal stimulation are applied simultaneously to symmetrical brain regions, which can lead to more complex effects [41]. tDCS can yield synergistic effects under certain conditions, such as enhancing cognitive balance or alleviating specific neuropathological conditions [51]. A recent study conducted demonstrated that applying left anodal/right cathodal and left cathodal/right anodal stimulation to the DLPFC led to a significant decrease in the amplitude of slow brain waves (δ, θ, and α waves) in the frontal, occipital, and parietal regions [51]. This is very consistent with our findings. However, it is important to recognize that the effects of tDCS on EEG power can vary among individuals. Additionally, research findings may differ depending on the duration and intensity of the stimulation [52]. In the present study, another significant finding regarding the impact of bilateral tDCS on the target region’s EEG was that cathodal stimulation of the right primary motor cortex led to a significant decrease in β power. The β power in the primary motor cortex is closely linked to motor and cognitive processes, characterized by a distinct “β rebound” phenomenon occurring before and after motor execution [53]. The activity of β in M1 reflects the neuronal state during motor control and preparation. Furthermore, during motor skill learning, β wave activity in the primary motor cortex undergoes changes. As skills are acquired and motor performance improves, the patterns of β wave activity may show adaptive adjustments, reflecting the brain’s neural plasticity. Therefore, the significant difference in β activity between the tDCS group and the sham group after training is an important indicator of more rapid skill acquisition.

Several limitations should be considered when interpreting our findings. First, due to a limited number of female participants, we were unable to achieve gender balance in our sample, leading to an all-male cohort. Future studies should address this limitation by specifically recruiting female participants to investigate potential gender differences. Second, our blinding assessment relied solely on post-experiment subjective reports from participants. A more rigorous approach would have included a systematic approach to guessing group allocation by participants to better evaluate the efficacy of blinding. Third, the sample size of this experiment was relatively small, which may impact the expected results. Finally, due to constraints related to experimental conditions and equipment availability, we were unable to conduct EEG recordings during the retention tests, which prevented us from conducting a neurophysiological assessment of the effects of tDCS on skill retention.

## Conclusion

This study suggests that applying bilateral primary motor cortex tDCS can enhance the acquisition of astronauts’ manual RVD skills and improve their retention. Considering the limited time available for each astronaut to train, along with the stringent requirements for skill mastery, these findings could accelerate the skill training process for astronauts. Given its established safety profile and ease of practical implementation, tDCS may represent a promising neuroenhancement tool for optimizing and maintaining astronaut training outcomes.

## Supplementary Information

Below is the link to the electronic supplementary material.

Supplementary Material 1.

## Data Availability

No datasets were generated or analysed during the current study.
