# Supplementary material for: Enhancing astronaut training: effects of transcranial direct current stimulation on manual rendezvous and docking skill acquisition
Source: J Neuroeng Rehabil. 2025 Oct 27;22:222. doi: 10.1186/s12984-025-01737-2 (PMC12557966; doi:10.1186/s12984-025-01737-2)
Supplement: Supplementary file 1 — Supplementary Material 1. [file 12984_2025_1737_MOESM1_ESM.docx]

**Table S1. Summary of Training Performance Data**

|  | **Position accuracy** | **Attitude accuracy** | **Propellant consumption** | **RVD score** | **NASA-TLX** |
| --- | --- | --- | --- | --- | --- |
| **tDCS group** | |  |  |  |  |
| 1^st^ block | 0.836 (0.411) | 6.343 (5.25) | 12.05 (4.749) | 49.694 (19.73) | 68.917 (12.588) |
| 2^nd^ block | 0.562 (0.27) | 3.304 (1.327) | 12.288 (6.066) | 61.878 (13.936) | 66.583 (13.256) |
| 3^rd^ block | 0.557 (0.28) | 3.7 (2.173) | 11.127 (3.917) | 62.899 (11.134) | 64 (13.253) |
| 4^th^ block | 0.437 (0.3) | 1.889 (0.843) | 11.523 (3.843) | 68.818 (9.202) | 62.333 (13.647) |
| 5^th^ block | 0.409 (0.268) | 2.103 (1.325) | 10.972 (3.73) | 69.81 (10.424) | 59.417 (14.786) |
| 6^th^ block | 0.477 (0.25) | 2.407 (0.857) | 10.794 (4.786) | 67.931 (12.219) | 57.333 (14.512) |
| 7^th^ block | 0.434 (0.249) | 2.34 (0.7) | 9.226 (3.208) | 71.404 (10.035) | 54.333 (13.173) |
| 8^th^ block | 0.433 (0.208) | 2.632 (1.429) | 10.604 (4.681) | 68.706 (10.995) | 52.667 (13.358) |
| **sham group** | |  |  |  |  |
| 1^st^ block | 0.755 (0.189) | 6.932 (3.009) | 13.695 (4.036) | 47.667 (11.065) | 70 (6.276) |
| 2^nd^ block | 0.543 (0.334) | 4.207 (3.081) | 10.669 (4.116) | 62.801 (14.174) | 66 (7.504) |
| 3^rd^ block | 0.505 (0.302) | 3.846 (2.854) | 10.98 (3.961) | 63.923 (15.466) | 64.214 (8.693) |
| 4^th^ block | 0.32 (0.234) | 1.83 (0.974) | 8.852 (1.972) | 75.569 (7.039) | 61.214 (7.587) |
| 5^th^ block | 0.331 (0.198) | 1.948 (1.209) | 9.879 (4.114) | 73.497 (12.257) | 58.357 (8.243) |
| 6^th^ block | 0.225 (0.082) | 1.353 (0.636) | 10.237 (3.49) | 76.544 (6.307) | 57.071 (8.722) |
| 7^th^ block | 0.151 (0.059) | 1.138 (0.417) | 7.486 (2.21) | 82.819 (4.109) | 55 (8.54) |
| 8^th^ block | 0.267 (0.131) | 1.678 (0.636) | 8.201 (2.354) | 78.047 (6.317) | 52.571 (9.163) |

All data reported as mean (SD)

**Table S2. Summary of skill retention test data**

|  | **tDCS group** | **sham group** |
| --- | --- | --- |
| Position accuracy | 0.217 (0.546) | 0.607 (0.129) |
| Attitude accuracy | 1.221 (0.801) | 2.834 (2.385) |
| Propellant consumption | 10.416 (4.496) | 8.249 (3.525) |
| RVD score | 72.966 (3.843) | 55.502 (11.191) |

**Table S3. EEG power statistics results**

|  | **Training** | | |  | **Group** | | |  | **Training *Group** | | |
| --- | --- | --- | --- | --- | --- | --- | --- | --- | --- | --- | --- |
|  | ***F*** | ***p*** | ***η^2^*** |  | ***F*** | ***p*** | ***η^2^*** |  | ***F*** | ***p*** | ***η^2^*** |
| **Left M1** | |  |  |  |  |  |  |  |  |  |  |
| theta | 5.910 | 0.025 | 0.228 |  | 0.104 | 0.751 | 0.005 |  | 4.151 | 0.055 | 0.172 |
| alpha | 2.241 | 0.150 | 0.101 |  | 0.847 | 0.368 | 0.041 |  | 0.038 | 0.847 | 0.002 |
| beta | 0.229 | 0.367 | 0.011 |  | 0.749 | 0.397 | 0.036 |  | 0.853 | 0.367 | 0.041 |
| total | 2.800 | 0.110 | 0.123 |  | 0.751 | 0.396 | 0.036 |  | 1.176 | 0.291 | 0.056 |
| **Right M1** | | | |  |  | | |  |  | | |
| theta | 8.576 | 0.008 | 0.300 |  | 0.250 | 0.623 | 0.012 |  | 5.410 | 0.031 | 0.213 |
| alpha | 0.001 | 0.982 | 0 |  | 1.328 | 0.263 | 0.062 |  | 0.522 | 0.478 | 0.025 |
| beta | 0.433 | 0.518 | 0.021 |  | 2.638 | 0.120 | 0.117 |  | 2.853 | 0.107 | 0.125 |
| total | 1.104 | 0.306 | 0.052 |  | 1.781 | 0.197 | 0.082 |  | 0.532 | 0.474 | 0.026 |
